# Personalised and Sustainable IEQ Monitoring: Use of Multi-Modal and Pervasive Technologies

**DOI:** 10.3390/ijerph20064897

**Published:** 2023-03-10

**Authors:** Graham Coulby, Adrian K. Clear, Oliver Jones, Alan Godfrey

**Affiliations:** 1 Department of Computer and Information Sciences, Northumbria University, Newcastle upon Tyne NE1 8ST, UK; 2 School of Computer Science, University of Galway, H91 TK33 Galway, Ireland; 3 Department of Technologies, Ryder Architecture, Newcastle upon Tyne NE1 3NN, UK

**Keywords:** indoor environmental quality (IEQ), personalised assessment, multi-modal monitoring, sensors, internet of things (IoT), building occupants

## Abstract

Background: Monitoring indoor environmental quality (IEQ) is important to better understand occupant health. Passive IEQ monitoring with digital technologies may provide insightful quantitative data to better inform, e.g., health interventions. Yet, many traditional approaches with known IEQ technologies have limited utility due to high costs or coarse granularity—focusing on the collective rather than individuals. Equally, subjective approaches (e.g., manual surveys) have poor adherence (i.e., are burdensome). There is a need for holistic IEQ measurement techniques that are sustainable (affordable, i.e., low-cost) and personalised. Here, the aim of this case report is to explore the use of low-cost digital approaches to gather personalised quantitative and qualitative data. Methods: This study deploys a personalised monitoring approach with IEQ devices coupled to wearables, weather data, and qualitative data, captured through a post-study interview. Results: The mixed-method, single-case approach gathered data continuously for six months with a reduced burden, by using digital technologies to affirm environmental factors, which were subjectively evaluated by the participant. Quantitative data reinforced qualitative data, removing the need for generalising qualitative findings against a collective. Conclusions: This study showed that the single-case, mixed-method approach used here can provide a holistic approach not previously obtainable with traditional pen-and-paper techniques alone. The use of a low-cost multi-modal device linked with common home and wearable technology suggest a contemporary and sustainable IEQ measurement approach that could inform future work to better determine occupant health.

## 1. Introduction

Poorly ventilated indoor environments can cause concentrations of a wide range of contaminants, off-gasses, and other substances, which are harmful to respiratory systems [1,2,3]. Their impact can negatively affect the health (and wellbeing) of building occupants [4,5,6]. Monitoring indoor environmental quality (IEQ) can provide a deeper understanding of conditions that can affect health, but this is complex and requires data on a multifaceted range of quantitative and qualitative outcomes. Typically, IEQ monitoring is costly/expensive [7], meaning measurements are either omitted completely or limited sensing modalities are adopted (i.e., uni-modal), which are often measured from a single spatial point. The latter means poor spatial density (of IEQ data), resulting in a one size fits all approach when trying to understand the indoor conditions experienced by individual building occupants [7], i.e., when the building is the unit of analysis rather than the individual. Accordingly, there is often a disconnect between what is measured by IEQ equipment and what is experienced by occupants.

Most IEQ monitoring studies focus solely on subjective data alone [2], which may be captured by surveying a sample of building occupants and generalising the findings for all occupants of a building [8]. This can be problematic for multiple reasons, as respondents may be inclined to respond according to accepted social norms or retrospectively backfill data after failing to complete surveys on time [9]. Surveys also inherently generalise findings to a population. For example, the ASHRAE Standard 55 [10] outlines that the pool of occupants surveyed should be large enough to ensure subjective biases do not form a general assessment of a building. Reducing subjective biases is important when the building is the unit of analysis, but when focusing on how individuals are affected by a building or how they respond to environmental change, the dilution of subjective biases results in a lack of individualism.

A notable challenge with traditional pen-and-paper questionnaires or surveys is the burden it can place on respondents. For point-in-time studies this may be less problematic as the survey becomes a one-time activity. For longitudinal monitoring, regular surveying can quickly become a burden, resulting in reduced participant adherence. Thus, longitudinal capture of individualised IEQ could benefit from contemporary, digital approaches that reduce burden by disrupting and/or supplementing traditional approaches with the multi-modal capture of environmental conditions. However, this requires monitoring environmental conditions that are local to the individuals under assessment.

## 2. Background

A body of work [7,8,11,12,13,14] has been undertaken by the authors here to explore solutions for IEQ monitoring that are scalable enough to support the monitoring of environments that are local to individual building occupants. A need for monitoring that focuses on the individual was identified [7], finding that individualised measurements could not only identify how individual occupants respond to environmental changes, but also that they could increase the spatial density of measurements, as numerous personalised IEQ monitors could be placed within a multi-occupied space. (Readers are directed to [7], which provides a comprehensive literature review on technological approaches to environmental monitoring—identifying a core knowledge gap around longitudinal monitoring in buildings that focuses on individual occupants). A previous study [14] was also conducted that explored technological approaches for monitoring individual building occupants that are affordable/sustainable. That study proposed and validated a multi-modal IEQ device [14] capable of gathering data on a wide range of environmental outcomes (including temperature, humidity, carbon dioxide (CO_2_)_,_ equivalent carbon dioxide (eCO_2_), total volatile organic compounds (TVOC), particulate matter, light and noise). That affordable, multi-modal approach could be an improved methodology to better monitor occupant health. To add further individualised focus, the use of wearable (physiological) sensors has also been identified [7] as complementing IEQ sensor measurements. Here, the ubiquity of affordable wearable health monitors is deemed as an opportunity, as they have the potential to reinforce qualitative approaches for IEQ data capture [5,7,9,15]. The combined technological approach could help link building occupants with the built environment to better understand the impact environmental changes have on individuals.

This case report aims to explore and understand whether personalised environmental sensing approaches can be used to address the subjectivity around environmental perceptions by providing quantitative context to how occupants experience building environments and whether the triangulation of data from the immediate environment, wearables and surveys can provide richer context compared to that traditionally found in environmental analyses. This study will implement a mixed-method approach for using affordable digital technologies to gather personalised, multi-modal data on environmental and physiological conditions in tandem with traditional qualitative data capture. A suitable methodological framework was adopted and adapted for making the individual the unit of analysis in an n-of-1 context, longitudinally monitoring a single participant using data from localised IEQ sensors, wearables, surveys, and interviews [8]. The n-of-1 methodology involves repetition around the measurement of a single individual over long periods of time, but since n-of-1 research methods focus on a single participant only, this is often mistakenly perceived as a limitation that results in resistance in practice [16]. However, n-of-1 methods are commonly used in medicine, psychology, psychotherapy, and special education [17], and are a valuable instrument for exposing time differential phenomena and causal links between measured outcomes [18]. For the purposes of this study, their use is novel and could potentially expose unique insights into the relationships between occupants and indoor environments at an individual level.

n-of-1 methods could potentially better link occupants to their environments to provide additional context to building analyses, by exploring, e.g., causal relationships between environmental outcomes, or by exploring recognised relationships between, e.g., ventilation and occupancy [19,20], as poor ventilation can cause contaminants to concentrate in indoor spaces that can lead to degradation in IEQ [21,22,23]. This can often be attributed to carbon dioxide (CO_2_), as CO_2_ can serve as a proxy measurement for ventilation [20], which can result in significant associations between CO_2_ and health, whereby CO_2_ is often reported as the main cause of building-associated sickness [24].

Since this study undertook longitudinal localised measurements, an additional consideration was taken here to ensure that the investigated approaches are affordable/sustainable, as state-of-the-art technologies have been found to be too expensive to be feasibly deployed at an individual level [7]. It is hoped that this approach could improve/enrich traditional approaches for IEQ monitoring to better understand the effects of IEQ on the health and wellbeing of individual building occupants, without generalising findings among a population of occupants.

## 3. Methods

**Figure 1:**
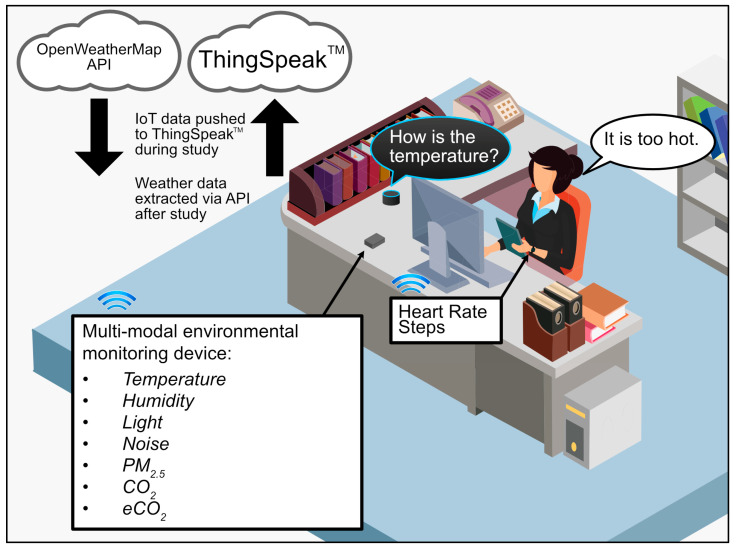
Diagram showing the general monitoring environment of the study including passive environmental sensors, wearables, voice-assisted surveys, and cloud platforms.

Due to the multi-modal nature of this exploratory study, a range of IEQ factors and associated outcomes (in parenthesis) were captured from a single low-cost device (approximately $50 per unit [14]) in each setting (Figure 1), namely: air quality (carbon dioxide: CO_2_, equivalent carbon dioxide: eCO_2_, particulate matter at 2.5 microns: PM_2.5_), temperature (degrees Celsius: °C), light (intensity: illumination/lux), humidity (percentage: %) and noise (levels: decibels/dBA). The environmental monitoring devices were developed and validated by the authors in a previous study [14] and were deployed within two different environments (buildings) common to the same (n-of-1) participant, a home and office setting. In brief, the devices comprised a Wi-Fi-enabled microcontroller (Heltec WiFi Kit 32) with a multitude of attached sensors (CO_2_: Winsen MH-Z19; particulate matter ≤ 2.5 microns in diameter, PM_2.5_: Plantower PMSA003i; eCO_2_: AMS CCS-811; temperature, relative humidity and air pressure: Bosch BME280; light intensity: ROHM BH1750; and noise: Invensense INMP441) to enable the multi-modal capture of key IEQ outcomes from a small-form-factor device [11]. All data were captured at 0.025 Hz [8] (i.e., 40 s intervals), but it should be noted that noise data were captured at the same frequency as other measurements taken by the device (continuous recording of audio was not conducted, and only decibel readings were captured and stored for ethical/privacy reasons). For the purposes of this exploration, wearable-based data were defined as physiological-based health outcomes, including steps (count: steps/minute) and heart rate (beats/minute: bpm).

### 3.1. Study Setting

The study location was a residential property in the Northeast, UK. The property contained two buildings (a home and garden-based office) within the same property boundaries (Supplementary Material Figure S1). During a pre-study examination of the entire property, some observations arose that were noted, and corresponding hypotheses were detailed as part of this exploratory study.

#### 3.1.1. Home

A semi-detached dwelling constructed pre-1950. The total floor area for the property was <100 m^2^ and one IEQ device was placed in the living room (25 m^2^). The property was brick-built with cavity walls, but no information could be obtained about the presence or thickness of cavity wall insulation. The roof was a pitched, tiled roof with 100 mm of insulation in the loft space. Accordingly, an arising hypothesis was posed (Table 1). The house/room was heated with central heating, which was manually controlled by a thermostat located in the living room. The room also had a gas fire in an existing fireplace, which was flued through an existing chimney. There was no air conditioning in the property, so natural ventilation was typically used for cooling. The home was entirely double-glazed and the windows for the living room were west-south-west facing. The room had several small lamps, but the primary light source was an artificial light source in the centre of the room.

#### 3.1.2. Office

The office was single occupancy, in a converted summer house, located at the end of the garden and situated near trees. The building was raised from the garden by a height of 1 m on a decked platform and was constructed of 23 mm walls, which comprised 15 mm solid timber panels and 8 mm of internal insulation. The roof was constructed of 19 mm tongue-and-groove boards on timber beams, with a green mineral roofing felt on the outer surface and no internal insulation. The floor was constructed with 19 mm tongue-and-groove boards on timber beams and was carpeted with 9 mm underlay. In the office there was an electric heater that was manually controlled to provide additional heat. However, there was no air conditioning, so natural ventilation was used for cooling. The office had east-facing doubled-glazed windows and a French patio door, i.e., the east side of the building had a large proportion of glazing (Table 1). The office had no blinds, so the participant was unable to block out light. There was a manually controlled artificial light source in the centre of the room.

### 3.2. Geographical Data

The openweathermap application programming interface (API) provides data on pollution and weather via supplied latitude and longitude coordinates, which are mapped to the nearest weather station. Using the current weather API, the nearest weather station to the study location was chosen. The station was <5 miles away from the study but is located near a major traffic route. Thus, it was not possible to determine the absolute accuracy of outdoor pollution for the given study location from the weather API. However, it is reasonable to assume that the weather data (e.g., temperature, humidity) obtained from this API was unlikely to deviate greatly over the distance between the dwelling and station. Hourly weather/pollution data were extracted from the OpenWeatherMap.org API using a free-tier account, which provided a quota of 60 calls/s, (60 geolocations/h). The free quota was suitable since data were only updated once per hour. To merge weather data with IEQ data, they were interpolated to replicate the hourly reading for each minute within the hour (e.g., an hourly reading of 24.6 °C reading at 12:00 would be copied to all values for that hour, i.e., 12:01, 12:02 … 12:59).

### 3.3. Ethics

Ethical consent was granted by the Northumbria University Research Ethics Committee (REF: 20481, 11 November 2019). The participant was a university employee and gave informed written consent before participating in this study.

### 3.4. Protocol

Due to the individualised approach, a suitable protocol was adopted which outlines a methodological framework (including multi-modal approaches with wearables) for monitoring individuals and their IEQ [8]. However, that protocol is a template only and must be suitably adapted for the needs and requirements of a participant.

### 3.5. Sample Size and Participant Details

As detailed in the protocol [8], this study focuses on the longitudinal investigation of a single participant in an n-of-1 context. Therefore, a single participant was selected for this study. The criteria for the selection were that the participant was of working age, was primarily an office-based worker and that they had a dedicated home office (this was a necessary criterion due to the COVID lockdown restrictions preventing work-based assessments).

### 3.6. Adapting the Protocol

An initial 1 h meeting was conducted with the participant to understand their expectations and to discuss the methods of data collection. Placement of quantitative IEQ devices, qualitative survey data capture and participant involvement were discussed. In the initial meeting, the participant was presented with an opportunity to tailor the protocol with the researcher to ensure that the study had minimal disruption to their work and home life/routine. This led to several protocol adjustments:

1. To ensure the participant was present at the point of data collection, they requested data be collected on weekdays only, during office hours (between 08:00 and 18:00). While passive quantitative sensor data were continuously collected from the home and office to compare, the office hours measurement window was deemed the focus of the study.
2. The participant also requested that the daily survey capture was conducted using a digital voice assistant to minimise the disruption to daily activities (See: Section 3.8.1).
3. The wearable was not worn at night due to charging requirements (See: Section 3.7)

### 3.7. Wearable

The wearable used in this study was the participant’s own Apple Watch Series 3. The participant explained that they did not find the watch comfortable to wear when sleeping, so they used this time to charge the device, as required daily. Accordingly, it was not possible to capture sleep data. As identified in the protocol [8], the participant was given instructions on how to export their Apple Watch data using the iOS Health app. A custom, client-side, web-based application [25] was then used to process and anonymise the health data. These data were processed by the application, which anonymised the data and provided the participant with the option to select the outcomes they want to submit to the study (all non-anonymised data were processed by the participant, locally on their computer and no data were stored by the application or transmitted to the cloud).

### 3.8. Qualitative: IEQ Perceptions

Two approaches were used in this study for measuring IEQ perceptions, which explored and compared contemporary approaches using digital voice assistants against traditional pen-and-paper approaches within interviews.

#### 3.8.1. Contemporary Data Capture: Digital Voice Assistant

The first approach for capturing qualitative data on IEQ perceptions involved capturing the participant’s ability to evaluate real-time environmental conditions. The participant felt that manual data entry (paper and/or digital) surveys would be disruptive to their work and habitual routines. Therefore, the participant requested surveys be deployed audibly using a digital voice assistant. Accordingly, the surveys were conducted using Alexa via an Amazon Echo, which was selected as the participant had experience using the Amazon platform. In line with their requests, an Alexa Skill was created using VoiceFlow [26], which is an online, web-based visual scripting tool for creating voice enabled applications for smart assistants, e.g., Amazon, Google. 

The Alexa Skill enabled voice-controlled events for (i) completing the survey or (ii) halting the survey if the participant was busy. The development of the skill involved creating logic blocks that could be chained together to create a program (Supplementary Material Figure S2). Loops and conditions can be used to create conditional flows and they can trigger Amazon Alexa’s text-to-speech engine to voice commands to the user. Microphone capture nodes can be used to listen to voice samples for keywords known as intents, which can be captured and stored as variables in Google Sheets. Of note, it is important to fully specify which intents the capture node should listen for to ensure there are no intent conflicts. For example, when testing this application, the question “How is the humidity?” could be answered freely without intent specification as the responses “too dry”, “too humid” or “comfortable” did not conflict with Alexa. However, for sound and light answers, “too light” or “too loud” would not be captured. Given that one can control light and sound on the Alexa device with voice intents, it was assumed that these were reserved keywords. By specifying that a capture node should expect these responses, Alexa allows the skill to use those keywords in that instance. Readers are directed to the study protocol [8] for more details on the survey responses.

#### 3.8.2. Traditional Survey/Interview Data Capture

The second approach involved using a modified version of the ASHRAE Standard 55 thermal environment satisfaction survey (TESS) [10], to align with current practice. To capture these data, an interview was conducted at the end of the study period, where the participant was asked a series of questions from a modified version of the TESS. Modifications were made to the TESS so that questions could be asked about the outcomes under assessment (temperature, humidity light, noise and air quality) but framed to follow the same format as the TESS. In line with the TESS, the participant was also asked when each issue was most prevalent (morning, mid-day, afternoon, evening, night). 

Since TESS prefixes thermal comfort questions with the following statement: “Please respond to the following questions based on overall or average experience in the past [six] month”, a further modification was made to interrogate “during the study period” as opposed to “in the past [six] month”. Since the TESS data focus on retrospective perceptions of IEQ, this study explored the data at a macro level, exploring averaged data against the retrospective qualitative assessments. Averaged, objective IEQ sensor outcomes were compared against (i) the qualitative data captured from the end-of-study interview and (ii) the quantitative data from the weather API and wearable device. Averages were captured for round-the-clock measurements, but the focus was on occupied hours during the analysis. Additionally, an outline of the analysis and statistical methods is presented.

### 3.9. IEQ Data Synchronicity and Acquisition

While the multi-modal IEQ device captured all IEQ data at the same frequency, not all data were synchronised, nor were the measurements taken from the wearable or the weather API. Synchronisation was needed for parity during any like-for-like comparison (for initial investigation and visualisation). For synchronisation, data were resampled to 1 min intervals—in line with a previous approach [14].

To collect, store and aggregate data, a cloud, internet-of-things platform called ThingSpeak^™^ was used, which was identified as being fit-for-purpose for real-time, multi-modal capture of low-frequency IEQ data [11]. Data were downloaded from ThingSpeak^™^ and labelled for easy identification. Data from the two IEQ devices (home and office) were then merged into a single CSV file using the timestamp (generated by ThingSpeak^™^, when data were sent from sensors) as the common field to join the two datasets. In short, each of the files were parsed from CSV format into a Pandas DataFrame, which was then resampled according to the frequency of the initial data capture.

**Figure 2:**
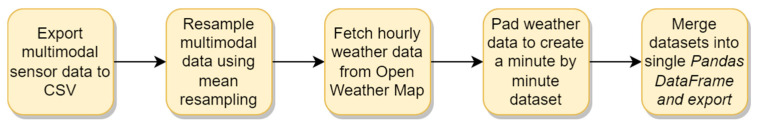
Multi-step process for merging data from multiple sources.

A multi-step process was used to create a single dataset suitable for conducting an exploratory analysis. Since the multi-modal sensors sampled every 40 s, mean resampling was used to create a dataset for both sensors with the resulting dataset containing fewer cases than the original sets, but with each newly created case equating to the mean value for each minute (Figure 2). In line with the protocol [8], these processes allowed for a complete dataset to be generated where missing data were accounted for, since missing data can cause biases in n-of-1 results [27]. Interpolation and resampling also aided bivariate analyses, as cases did not need to be removed where missing data were present.

### 3.10. Quantitative: Wearable

Data were extracted from the wearable using a bespoke application/app [25]. The app created individual CSV files for each outcome, which were processed before analysis to ensure they could be analysed in the same context as the IEQ data. For example, the step count was recorded by the Apple Watch for a given period of sustained activity/walking. To enable minute-by-minute comparison, data were divided into 1-min bins, e.g., 50 steps for 12:05 and 12:06, then 25 steps for 12:07. Where no steps were recorded for a given minute, those data were padded with a 0. Like step events, HR was also captured at random times. Since Apple Watch heart rate measurements are frequent, but not continuous, heart rate data were recorded with variable measurement increments. For example, on occasion, multiple heart rate measurements were taken in 1 min, followed by gaps in the measurements between 2–20 min.

While it was possible to pad measurement intervals in the steps data with zeroes, as step events would only be recorded after a bout of steps, the same could not be done with HR. This is because 0 bpm would not be a valid measurement of heart rate. To remedy this, interpolation of heart rate was required to fill in the data missing due to measurement intervals. 

### 3.11. Interpolation of HR Data

Linear interpolation methods produced a valid range in the data but resulted in an unrealistically smoothed dataset. In contrast, non-linear interpolation contained values that were invalid due to not only being significantly outside the extrema of the original data, but were outside the extrema of acceptable (e.g., −50 bpm). To overcome unrealistic values, a piecewise cubic Hermite interpolating polynomial (PCHIP) was used, which produces polynomial interpolants that are bound by the extrema of the original data [28]. Since PCHIP interpolants were representative of existing data, the interpolants between two points were restrained by those points. This provided that anomalous events would not be generated by the process. However, this also meant that transient events, such as sudden spikes/drops in the heart rate would not be captured. However, since the Apple Watch would record transient events and create a measurement for such instances, this was not deemed a concern.

### 3.12. Visual Analysis

A combination of SPSS and Excel (v2112, Microsoft) were used to generate pivot tables, graphs, and statistics. This enabled broad descriptive statistics (minimum, maximum, mean values, and standard deviation) and visualisations. Bivariate analyses of data pairs were generated from SPSS, so that key data pairs could be evaluated. The primary statistical approach for this paper is visual analysis, conducted by graphing variables for comparison over averaged time frames.

### 3.13. Data Validation

An initial check was conducted that confirmed that the sensors were reporting data within the expected range of the sensors and were performing within the expected norms according to their evaluation in a previous validation study [14] (Supplementary Material Table S1). This check also identified the scale of missing data. The process of collecting data from the weather API and the Apple Health Data Parser meant data were already padded to include missing values; therefore, these variables had a maximum valid N (listwise) of 205,016. When collecting data from the multi-modal devices (home and office), those data were not interpolated, resulting in approximately 20% data loss.

## 4. Findings

To conduct the visual analysis of the outcomes, TESS responses were first explored to identify areas of interest within the data. Qualitative findings provided intriguing, yet subjective, points of view. These provided ‘windows’ into the data to act as a starting point within the visual analysis. To conduct the analysis, data were graphed to help visualise bivariate relationships, or links between qualitative and quantitative findings. This section will present each of the core outcomes that were explored in the TESS survey (temperature, humidity, light, noise and air quality).

### 4.1. Temperature

**Figure 3:**
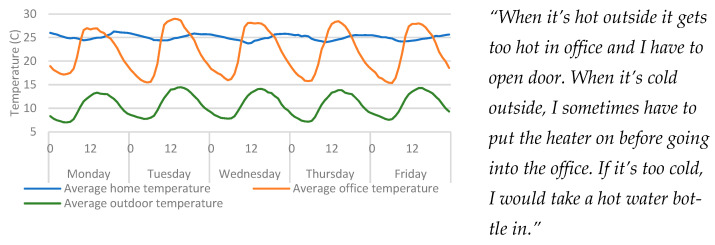
Average hourly temperatures (per weekday) from each temperature source (home, office, and outdoor). Qualitative findings are displayed alongside the quantitative data for context.

TESS-based qualitative findings for temperature indicate that there is a link between the office temperatures and outdoor temperatures (Figure 3). It was identified that the office building lacked sufficient insulation and it was hypothesised that the thermal conditions of the building would be impacted by outdoor conditions. By inspecting the data from the IEQ devices in the home and office against outdoor weather data (Figure 3), the temperature from the office IEQ device demonstrates a large degree of variation (range: 15 to 29 °C) during day and night cycles, and these readings strongly correlated with outdoor temperatures. When compared to the data from the home (range variation: 24 to 26 °C), this highlights that the office was significantly less insulated than the home. This also confirmed the hypothesis that the home should provide greater thermal stability due to the increased insulation.

As the participant highlighted, the source of heating between the two properties differed. While the home used central heating, controlled by a thermostat, the office heat was controlled by an electric heater. This heater was only used when the office was occupied and only when it was cold. Central heating systems work by using a heating element to heat water which flows through the radiators, and radiators can store heat after the heating source is turned off [29]. This could indicate why the temperature was dropping off so suddenly in the office, since the office was heated with an electrical element heater. However, since the home was controlled by a central thermostat, that kept the property at a given set-point temperature. However, as Figure 3 highlights, the temperature drops in the home were much less sudden than that of the office, further confirming the hypothesis around the home’s thermal stability.

### 4.2. Humidity

**Figure 4:**
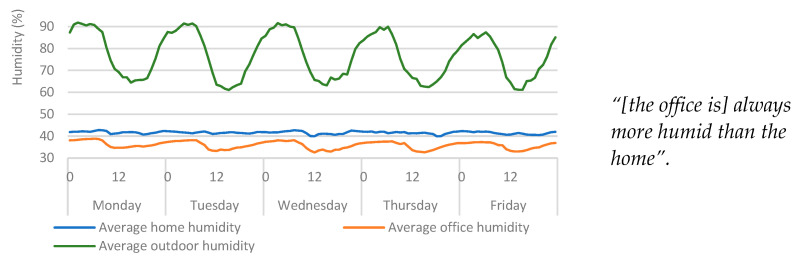
Average hourly humidity (per weekday) from each humidity source (home, office, and external). Qualitative findings are displayed alongside the quantitative data for context.

The participant felt that the office was always more humid than the home. However, the sensor data (Figure 4) imply the opposite. One possible explanation for this is that the human body is better able to manage core temperature within stable thermal environments and transient conditions can significantly affect thermal sensations [30]. This means that people are better able to provide qualitative assessment of the environment under stable thermal conditions. Given that qualitative assessment through self-reporting is one of the primary mechanisms for the assessment of building performance [2,7], this raises questions over the efficacy of these methods. Therefore, the lack of thermal stability in the office environment may be an influencing factor as to why the participant was unable to provide an accurate self-reported assessment of the humidity (in the office). These findings present the case for augmenting quantitative data into building performance and comfort assessment, to support and/or validate the findings obtained from subjective assessment. This comparison also provides further evidence of insufficient insulation in the office.

### 4.3. Light

**Figure 5:**
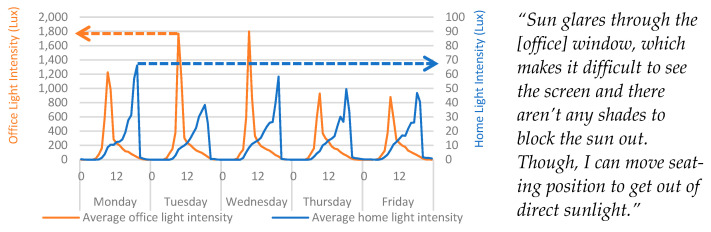
Average home/office light intensity categorised by day > hour. Qualitative findings are displayed alongside the quantitative data for context. Two vertical axes are used here due to scale differences; therefore, colour-coded arrows are used to highlight the scale used by each parameter.

The participant expressed a clear dissatisfaction with the lighting in the office, remarking that it was “often too light in the morning” and “always lighter than the home” (Figure 5). Figure 5 shows that the measurement ranges for each light sensor were vastly different and light in the office was consistently, significantly greater than that of the home. Figure 5 also shows that the office consistently measured high levels of light intensity during the morning period, which is in line with the findings from the qualitative data and with the expectations in the identified hypotheses.

It was also posed that geographical positioning was the primary cause of the reported phenomena. The window for the home environment faced due west-south-west, whereas the windows in the office faced due east. A sun map was created using Autodesk Revit (v.2022.2.1, Supplementary Material Figure S3) to track the sun movements during a randomly selected day during the study period. The sun map shows that after midday the sun would move behind the office and begin to cast light on the home. However, the shape of the home, and the treeline meant that the sun did not have a direct line-of-sight until around 18:00, by which time it was setting.

Light data from the IEQ devices show a demonstrable consistency with self-reporting data obtained from the qualitative assessment in this study and the modelled sun maps. The qualitative assessment did identify that the participant was dissatisfied with the light in the space, but it did not indicate the extent of the problem. Quantitative data, in this instance, were extremely useful for identifying times when the light intensity would be disruptive to work and the specific levels of light intensity. This presents a strong case for the longitudinal capture of localised, quantitative data in building performance and comfort assessments.

### 4.4. Noise

**Figure 6:**
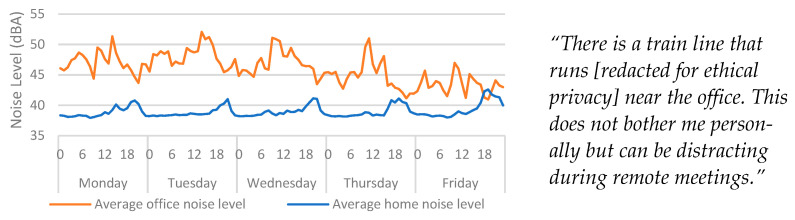
Average home/office noise levels categorised by day > hour for Monday to Friday. Qualitative findings are displayed alongside the quantitative data for context.

The participant explained that there was a train line that passed close to the office building (Figure 6). Due to the proximity of the railway, averaged data for sound were explored to see if high sound pressure levels (SPLs) were noticeable that may indicate the regularity of trains. However, the frequency of data capture (0.025 Hz) meant that this inquiry could not be observed at this level of interrogation. Figure 6 shows that the average noise levels were regularly between 5 dBA and 15 dBA louder in the office than in the home. Daily average trends also show that the highest average values in the office were always during office hours, indicating occupancy-related noise levels. Comparatively, the highest average values in the home were recorded in the evening, outside of office hours. This indicates that sound sensors could also serve as a proxy measure for occupancy. However, the use of audio recording equipment for detecting human occupancy could raise issues of ethics, privacy, and trust, whether voice is recorded or not. It is also worth noting that the noise level ranges in the home were within building standards (25–40 dBA), but the office noise ranges were more aligned to outdoor acoustic environments [31]. This further highlights that the construction of the office (lack of insulation) could also impact the acoustic properties of the space.

In this study, no audio was recorded, and sound pressure levels were measured at the same sample rate as other measurements captured by the multi-modal device (40 s). So, while it may be possible to detect occupancy, it would not be possible to understand spoken words or determine who was present in a space. If a greater sampling rate is required, it is important to consider the ethical implications this may have, especially if the resulting data could be deciphered in such a way where spoken words could be observed.

### 4.5. Air Quality

To obtain quantitative measurements of air quality, PM_2.5_ was used to measure the presence of dust, pollution and odours, and carbon dioxide was used as a proxy measure for ventilation and subsequently used to determine occupancy and air circulation.

#### 4.5.1. Particulate Matter (PM_2.5_)

**Figure 7:**
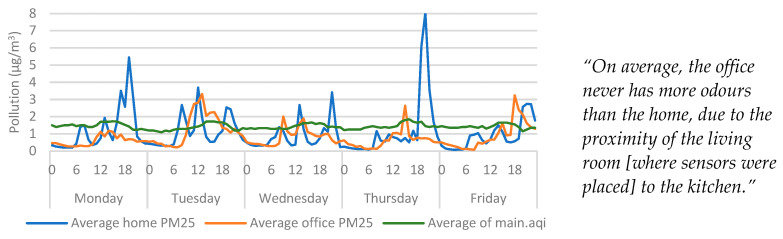
Average outdoor air pollution vs indoor particulates categorised by day > hour. Qualitative findings are displayed alongside the quantitative data for context.

The recorded highs for particulates within the home were consistent with mealtimes (Figure 7), which may indicate that that the sensor was affected by cooking from the kitchen which neighboured the living room. It also may explain the participant’s comments regarding odours in the property, as PM_2.5_ sensors can be highly sensitive to certain types of cooking, especially when frying or cooking with oils or fats [32], so this finding is in line with expectations. The highest averages were recorded typically around 18:00, which is consistent with when an evening meal would be cooked—based on the average time the participant left the office. Therefore, it is important to consider the proximity of PM_2.5_ sensors from pollution sources. While the data highlight links between cooking and PM_2.5_, Figure 7 does not indicate baseline values that would suggest the office was dustier than the home, nor did the PM_2.5_ averages indicate regularity in the data that would suggest correlations with timetabled train services. However, if the dust in the office was predominantly caused by airborne debris from passing trains, it is likely that the diameter of the micro particles was larger than the PM_2.5_ sensor could measure.

**Figure 8:**
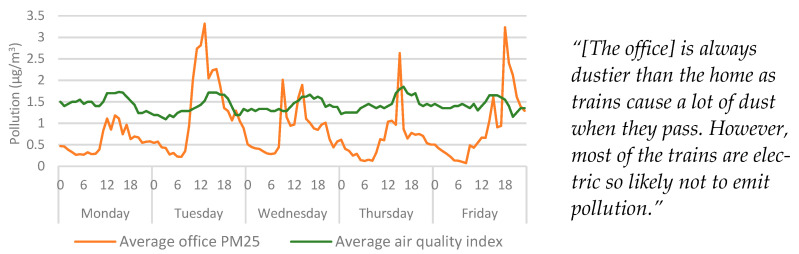
Average outdoor air pollution vs office particulates categorised by day > hour. Qualitative findings are displayed alongside the quantitative data for context.

The visual inspection of PM_2.5_ data showed that both the office PM_2.5_ and outdoor pollution increased around midday. Due to the scaling in Figure 7, the extent of this is not clear, but these trends can be seen more clearly when the home is removed from the graph (Figure 8). Since temperature and humidity were affected by poor insulation, it was expected that the indoor pollution could be influenced by outdoor conditions as well. However, the influence outdoor pollution had over indoor measurements was less significant than either temperature or humidity. However, it is possible that this was due to the distance between the study location and the weather station.

#### 4.5.2. Carbon Dioxide (CO_2_)

**Figure 9:**
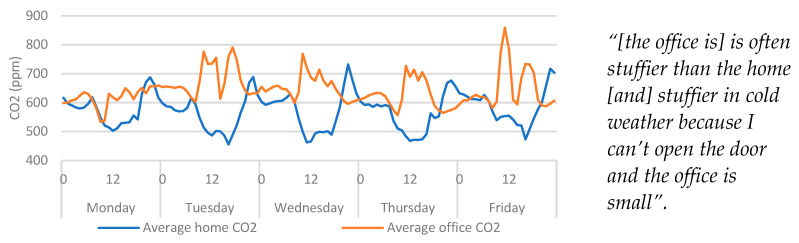
Average home/office CO_2_ categorised by day > hour. Qualitative findings are displayed alongside the quantitative data for context.

Qualitative data surrounding air circulation indicated that the home had better circulation than the office. Given that the home is slightly more than double the square meterage of the office, the participant’s comment on the circulation was not unexpected. Figure 9 shows that even during the evenings, when the home environment was occupied, the maximum average CO_2_ never exceeded that of the office despite the participant sharing that space with their partner, while being the sole occupant of their office.

Given that the participant felt that the room was stuffier when the door to the office was closed, it is worth exploring the links between occupancy and CO_2_ levels, as CO_2_ sensors are useful indicators of occupancy and can be used as a surrogate measurement to determine the level of ventilation within indoor environments [14,19,20]. As already seen in Figure 9, patterns can be observed that indicate transitions from the home to the office during office hours. During these times CO_2_ can be seen to increase in the office, while decreasing in the home.

### 4.6. Linking the Occupant to the Environment

Since CO_2_ is regarded as being the main cause of building-associated sickness [24], it is worth exploring the relationships between physiological responses and CO_2_. Since links between CO_2_ and working hours have already been observed here, occupancy and physiological responses will be observed from wearable data within the context of CO_2_ data.

#### 4.6.1. Occupancy Analysis

**Figure 10:**
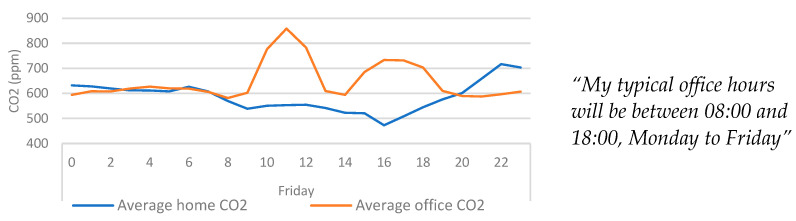
Average home/office CO_2_ categorised by day > hour for an average Friday. Qualitative findings are displayed alongside the quantitative data for context.

By inspecting the averaged CO_2_ data of a single day (Figure 10), it is possible to see transitions between the home and office more clearly. Inverse relationships between office CO_2_ and home CO_2_ can be observed, which are also aligned with office hours and the qualitative accounts of working hours provided by the participant. Interestingly, Figure 10 also shows that on average the CO_2_ shows a significant drop on Friday afternoons. This could indicate that the participant regularly left the office during these times.

**Figure 11:**
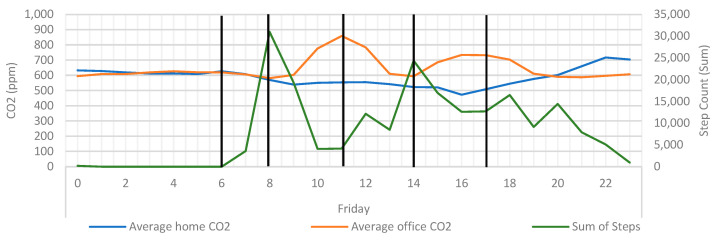
Average home/office CO_2_ overlayed with sum of steps and key event markers. Vertical black lines signify the point when CO_2_ rises or falls.

It was hypothesised here that if the CO_2_ levels observed here are indicative of occupancy, there could be a relationship between CO_2_ measurements and participant activity. To investigate this, step data were included and overlayed over the CO_2_ averages (Figure 11). In doing so, it was possible to see that there were a series of inverse relationships between the CO_2_ averages and summed step count. As the participant transitioned from walking to resting (step count rising and falling) CO_2_ levels in the office would begin to rise. Conversely, as the CO_2_ levels in the office began to fall, an increase in step count could also be seen. By plotting these events, it is possible to see events when the participant may have entered and exited the office. This demonstrates that the combination of step data and CO_2_ can be used to detect occupancy, but it is anticipated that this phenomenon can only clearly be observed in a single-occupancy space.

**Figure 12:**
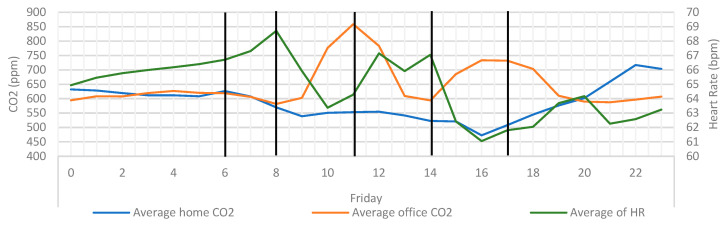
Average home/office CO_2_ overlayed with average heart rate and key event markers. Vertical black lines signify the point when CO_2_ rise or falls.

This link between CO_2_ and activity can be further affirmed by looking at the average heart rate for the same period (Figure 12). CO_2_ has been shown to rise when steps decrease, and fall when steps increase, and the same is true for heart rate. This would indicate that the CO_2_ being measured in the office is linked to the sedentary behaviour of the participant.

**Figure 13:**
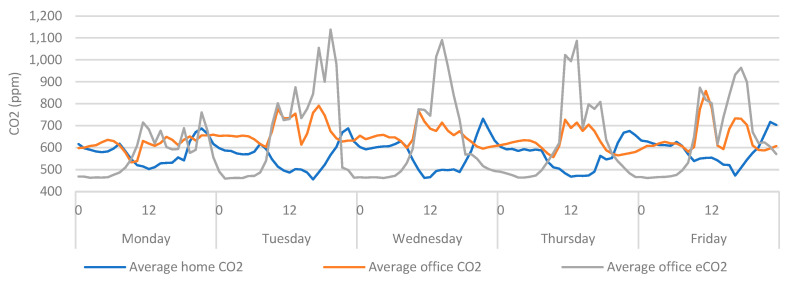
Average home/office CO_2_ categorised by day > hour.

These findings provide demonstrable evidence towards the value of including wearable data within environmental assessments, but they also provide further evidence towards the capabilities of CO_2_ sensors as proxy measurements for ventilation and occupancy. This has been identified in previous work [14], where metal oxide sensors were identified as having the potential to serve as a lower-cost, proxy measurement for ventilation, as they can measure the oxidation of a wide range of gasses (typically labelled as total volatile organic compounds (TVOC), or equivalent carbon dioxide (eCO_2_)), which can concentrate within the air in indoor spaces. Therefore, it is worth also exploring these data within this context [14]. By including the eCO_2_ measurements (from the office), similar trends (Figure 13) can be seen as those observed in previous work [14]. The eCO_2_ sensors provided more erratic measurements when compared to CO_2_, yet the measurements also provided a similar indicator of office-based occupancy. 

**Figure 14:**
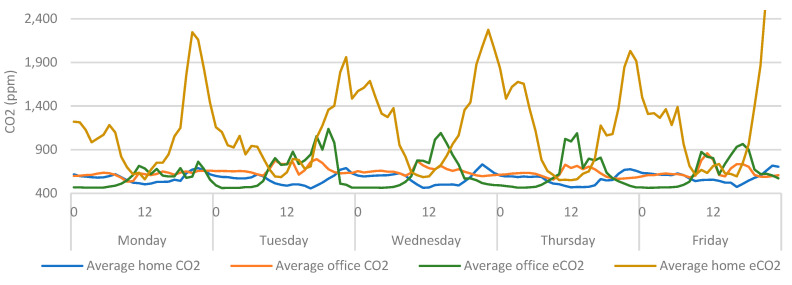
Average home/office CO_2_ vs eCO_2_ categorised by day > hour.

When the eCO_2_ data from the home is also included (Figure 14), the movement in eCO_2_ data becomes much more exaggerated. If this were used as a proxy measure for ventilation it would be safe to assume that the participant left the home at around 6:00 am but did not enter the office until 9:00. There is also a degree of crossover at around 15:00. Consequently, the same assumptions cannot be drawn from Figure 14 as can be drawn from Figure 10. Given that eCO_2_ sensors are highly sensitive to a wide range of environmental conditions, pollutants, and gases [33], the erratic behaviour of the sensor could indicate that the increased quantity of fixtures, fittings and furnishings *(present in a home environment)* are saturating the indoor eCO_2_ sensors with an increased concentration of airborne pollutants. Thus, these sensors may be less useful for providing a proxy measure of ventilation in environments with high concentrations of pollutants.

#### 4.6.2. Daily Voice Assisted Surveys

Throughout the 142-day study period the participant only successfully managed to complete 48 surveys, due to work commitments or forgetting to revisit the survey after delaying the data capture. This highlights that while this was the participant’s preferred method for daily data capture, there are persistent challenges surrounding participant adherence when active participant involvement is required in studies.

The limitations placed on the survey capture to reduce burden *(limited outcomes, three-point responses)* resulted in data that significantly lacked context. For example, the participant responded “too hot” when it was 14 °C in the office, but cold when it was 31 °C and responded comfortable between 17 °C–31 °C (Table 2).

The methods to reduce burden resulted in a lack of contextual data regarding, e.g., clothing, heating/ventilation status, times of meals/activities, sleep quality, hormonal/stress levels, etc. However, increasing the line of questioning to include such data would be more burdensome than traditional approaches. Despite the low adherence, and lack of context, the voice assistant survey capture mechanism became the focus of attention within the closeout interview, highlighting novel research themes for future inquiries in this field.

### 4.7. Supplementary Qualitative Data

The closeout interview provided the participant with the opportunity to provide feedback on their level of involvement, where the conversation became focused on the use of Alexa for survey data capture. The participant found the Alexa to be a preferable experience overall stating:

*“I almost created a little relationship with Alexa, which I presume was a sort of indirect relationship with you as the researcher, where it was like I’m going to do that thing today I’m going to do it for Graham for part of his survey”*

This was apparent while the participant was talking about Alexa, as they regularly personified the device making statements such as:

*“Occasionally, she would interrupt me in the middle of a meeting and ask is I was ready to do the survey.”*

The participant also went on to say that the experience of conducting the survey vocally allowed them to engage with the survey without having to switch tasks, reflecting on the personability of the device and the relationship with the researcher throughout:

*“[when asked if able to do survey during a meeting] I would say no to her, but I think the majority of the time I always made the effort to pick up on our survey and do it later. I don’t know if I would have done that if it had been an online or written survey, (…) partly because there was less effort involved on my part to fill the survey in, I could do it whilst I was doing other things, but also that relationship of talking to a voice almost created an incentive to not let that person down and complete that task right.”*

This provides an interesting angle on the problem of reducing the burden towards participants. The three-point assessment conducted daily was originally chosen to complement the TESS approach conducted at the end, to evaluate whether shorter surveys that quantified experience could reduce the burden experienced during longitudinal assessment. The participant highlighted that:

*“If I had to do an online questionnaire or written questionnaire every day, I would be pretty cheesed off by now, but now I am not as it has been quite an exciting process and I was discussing with my family yesterday saying, ‘Graham is coming round tomorrow, he’s been collecting my data’ and I was quite enthusiastic about it”*

This highlights that the method of data collection had a more notable reduction of burden on the participant than the reduction of responses within the survey. In fact, the participant stated:

*“If there was an option to add additional information, if you really wanted to, in a free-text way, that would have been useful because I could have said ‘it’s raining really heavily outside’ or ‘I’m not feeling well today’ (…) that will give you additional information (…) but also it does get a little bit annoying when you have got somebody repeating the same words to you every day.”*

This highlighted that the use of the three-point assessment made the participant feel like they could not provide justifications as to why they were responding in a particular way. They expressed desire throughout for a natural language system, where their responses did not need to be scripted, allowing for qualitative data capture. This provides an interesting line of inquiry, as it could have provided a mechanism for the participant to provide the additional context required to add statistical value to the data being captured. However, this would also require conversational natural language processing that would exacerbate existing privacy concerns surrounding digital voice assistants [34]. Consequently, this would also require additional ethical governance and data management protocols since spoken voice recordings can be used both for biometric verification and for training artificial intelligences to impersonate a target to bypass verification [35].

## 5. Discussion

This study sought to investigate whether contemporary and affordable approaches for individualised IEQ monitoring can be used to address the subjectivity around environmental perceptions. If sensors are deployed longitudinally, with multiple sensing modalities, they can be used to address the subjectivity around retrospective environmental perceptions by providing quantitative context to how occupants experience building environments. The subjective, open-ended responses offered in the TESS format provide a valuable opportunity to gain insights on individual experience, without overloading participants with an unrealistic and burdensome line of questioning within longitudinal assessments. For the purposes of this paper, the TESS questions were rephrased from “in the past [six] months” to “during the course of the study”, so that the subjective responses gathered from the participant were based on generalised opinions over the course of the study and not based on any real-time, or point-in-time measurement, which is more aligned to the averaged data from quantitative measurements.

The qualitative data capture provided a useful mechanism for highlighting areas of interest within the longitudinal data. By focusing on the qualitative data first, the quantitative data could be explored in relation to qualitative findings. For example, the comparisons the participant highlighted regarding light intensity provided an interesting line of inquiry that could be explored using a combination of quantitative sensor data, knowledge of the study location and its buildings, and seasonal data. This inquiry not only provided quantitative affirmation of the participant’s perceptions, but it also highlighted the value of technologically enhanced qualitative data capture. By conducting the study as presented here, multi-modal quantitative data capture has demonstrable utility in supporting traditional qualitative capture methods. The findings show that the responses from an individual do not need to be categorised as in-or-out of a 95th percentile, but instead the opinions and perceptions of individuals can be treated as valid data for analysis, regardless of their subjectivity. This has practical implications as not only could this provide a more thorough understanding of how individuals respond to indoor environments, but it could also reduce the number of occupants required to assess a building (where the building is the unit of analysis). Localised monitoring could be used to remove subjective biases so that generalisation of qualitative responses is not required, thus preserving individuality in the data.

The use of wearables in this context provided a valuable mechanism to align the individual with the environment through synchronous measurement of environmental and physiological data. The wearable data provided additional context to the data captured from passive IEQ monitoring devices and they provided evidence of causal relationships between environmental changes and physiological responses. However, while the IEQ devices were able to monitor continuously, the Apple Watch 3 required regular charging cycles that meant it would not be possible to measure around the clock, even if the participant were to have worn it at night. Many personal fitness trackers (PFTs) have extended battery lives (>1 week) and short charging cycles (<1 h) meaning that they can be used to monitor health outcomes day and night. However, smart watches integrate many additional features, which increase adoption rates but significantly impact battery life, requiring daily charging. Since sleep was not defined as an outcome of this study, this did not impact the collection of data required for this investigation.

Overall, this study longitudinally observed a single participant between their home and office environments using n-of-1 methods. While n-of-1 methods are often perceived as a limitation [16], the longitudinality coupled with individuality of the data obtained created valuable context that would otherwise be unavailable with traditional measurement approaches. While their adoption in this study served as an exploratory first step into the use of n-of-1 methods in this domain, their value warrants further exploration in the field. However, there were specific deployment challenges and technical considerations that can impact longitudinal studies of this nature, that readers should be aware of.

### 5.1. Technical Considerations

#### 5.1.1. Addressing Problems in the Field

Visits to the study location beyond routine data collection were required. The first arose when a pet knocked the office based IEQ device off the table, damaging the microcontroller. This resulted in data loss for several days due to the need for the participant to be at home to enable access to the property. However, the IEQ devices were designed so components could be substituted in the field, enabling a speedy repair. Additionally, there were issues with SIM card connectivity throughout this study. Although contingencies were put in place (based on the lessons learned from a previous study [13]) to restart the sensors in the field (using smart power strips to remotely restart devices when they became unresponsive), the smart functionality of the power strips was halted in the event of a data outage. However, the participant was able to restart the 4G router by manually turning the smart switch off and on, which removed the need to make multiple visits to the study location. Due to the data outages, the study ran from 15 March 2021 until 4 August 2021 (>20weeks, 142 days) to ensure sufficient data were captured.

#### 5.1.2. Data Capture: Sampling Frequency

As identified in previous work [13,14], high-frequency sample rates could be problematic in a multi-modal device, such as the one proposed, as it would mean the processor of the microcontroller Unit (MCU) would potentially be continuously blocked by sound pressure level calculations. Multicore MCUs could enable high-frequency data capture and synchronous, e.g., SPL, calculations, but the processing bottleneck would be pushed to the networking functionality (WiFi/Bluetooth/BLE/Zigbee, etc.) instead, when data are transmitted to the cloud [14]. Moreover, to record this level of data, a large amount of storage would be consumed in a short space of time and bandwidth/messaging quotas would also be rapidly consumed. The 40 s sample period may not have been able to address specific lines of enquiry (relating to noise levels) at this stage of the investigation, but this data capture frequency meant that both the unit costs and the running costs of the multi-modal were low.

#### 5.1.3. Amazon Alexa

The skill was distributed using an Alexa Routine so that the survey prompt was delivered to the participant at 10:30 am Monday–Friday (in accordance with the participant’s request). The process of deploying the application to Amazon was inhibited by Amazon’s beta testing policy, which prevented long-term deployment of the skill to third-party users. Such limitations were not present for the developer account (the amazon account owner), so a dedicated Amazon account was setup whereby the participant was the developer, giving them full, uninhibited access to the skill. Amazon provides the capability for skills to be hosted privately and distributed in the same way as public skills. However, this feature is only available to business organisations. Therefore, small research groups may need to consider publishing the skill publicly if larger sample groups were recruited outside of business use. The VoiceFlow application also allows applications to be distributed to Google Nest voice assistants, which may not be as limiting. However, this was not explored within this study.

### 5.2. Limitations

This participant identified transport infrastructure as a potential source of pollution. However, data related to cars on the road, train timetables, engine types, etc., were not planned for nor used in this study. Given the findings, such outcomes could potentially be useful when conducting similar research. The inclusion of, e.g., traffic camera/timetabling data could be beneficial when exploring residential indoor air quality near transport infrastructures.

### 5.3. Future Research

This study has highlighted that personalised environmental sensing approaches can be used to address subjectivity around environmental perceptions, if the individual is the unit of analysis and the measurements are longitudinal in an n-of-1 context. This study focused specifically on a single participant to explore whether richer context could be gained from the participant, building or both. However, low-cost technologies were selected for this study to ensure that this research has pragmatic implications beyond this research. It is envisioned that future researchers could expand upon this research in two ways. Firstly, while n-of-1 methods are specifically designed for a single participant, tandem n-of-1 studies can be conducted to measure larger sample sizes to determine if individual findings are general to a population [17]. Secondly, future researchers could explore portable environmental monitoring devices that are capable of monitoring environmental conditions as wearers transition environments. However, it is envisioned that further research would need to be explored into the effect environmental transitioning (traveling from indoor to outdoor spaces) has on sensors.

## 6. Conclusions

This study conducted an exploratory, mixed-method investigation of personalised environmental monitoring, by triangulating data from surveys/interviews, environmental data, and wearables. In doing so, this paper was able to highlight the value in augmenting traditional data capture instruments with emergent technologies. The explored methods provided a useful mechanism to address subjectivity in qualitative data capture in building studies. Subjectivity is a natural expectation when evaluating perceptions, but this study outlines a means of elevating the opinions of individuals within the analysis, rather than generalising those opinions within a population. By making the individual the unit of analysis, qualitative data can be enriched by quantitative data from wearables, APIs and environmental sensors. The quantitative data provided direct affirmations of individual perceptions, while providing additional context that could not be achieved with traditional approaches of data capture, which largely focus on qualitative data alone due to the cost and complexity of traditional measurement equipment. This was enabled using validated, affordable (low-cost) emergent hardware, which provided a means to explore environmental changes local to an individual using localised sensors in an affordable/sustainable manner. Not only did this enable the results outlined in this paper, but this also highlighted a solution for localised environmental monitoring that is scalable and feasible outside of research environments. Thus, this paper provides a valuable extension to traditional methods of building performance assessments, which could be useful to future researchers and industry practitioners.

## Figures and Tables

**Table 1:** Arising hypotheses.

Observation	Hypothesis
The home had good build quality with appropriate insulation (i.e., insulated cavity walls and insulated loft space. In contrast, the office was poorly insulated (i.e., none in the floor or roof and a thin wall insulation layer only).	The home should provide a greater thermal stability when compared to the office.
The office had no blinds (i.e., window shades)	Due to the geographical positioning of the office, morning sunlight will saturate the office.

**Table 2:** Snapshot of participant’s perceptions towards temperature alongside indoor and outdoor measurements.

Date/Time (UTC) *	Perceived Temp	Office Temp (°C)	Outdoor Temp (°C)
2021-16-03 10:53	comfortable	28.66	4.62
2021-17-03 10:56	comfortable	19.08	7.14
2021-18-03 10:38	comfortable	17.81	7.86
2021-19-03 11:20	hot	26.76	7.70
2021-19-03 15:42	hot	28.72	8.37
2021-22-03 10:31	cold	18.23	3.52
2021-24-03 10:30	cold	26.68	6.73
2021-29-03 09:32	comfortable	21.67	7.70
2021-30-03 15:18	comfortable	26.80	11.23
2021-31-03 09:31	comfortable	28.20	6.07
2021-22-04 09:31	comfortable	32.84	5.32
2021-27-04 09:44	comfortable	31.18	6.10
2021-28-04 09:31	comfortable	22.33	7.86
2021-29-04 10:13	comfortable	25.45	11.63
2021-30-04 09:31	hot	14.30	7.67
2021-04-05 11:06	hot	31.73	9.81
2021-05-05 09:31	cold	31.08	6.04

* UTC offsets resulted in timestamps that were 1 h earlier than when the survey was conducted.

## Data Availability

The datasets generated during and/or analysed during the current study are available from the corresponding author on reasonable request.

## Supplementary Materials

The following supporting information can be downloaded at: https://www.mdpi.com/article/10.3390/ijerph20064897/s1.
