# Supplementary material for: Personalised and Sustainable IEQ Monitoring: Use of Multi-Modal and Pervasive Technologies
Source: Int J Environ Res Public Health. 2023 Mar 10;20(6):4897. doi: 10.3390/ijerph20064897 (PMC10049265; doi:10.3390/ijerph20064897)

Supplementary Material

Supplementary Material Figure S1. Study location detailing sensor locations, as well as light, heating and ventilation sources.

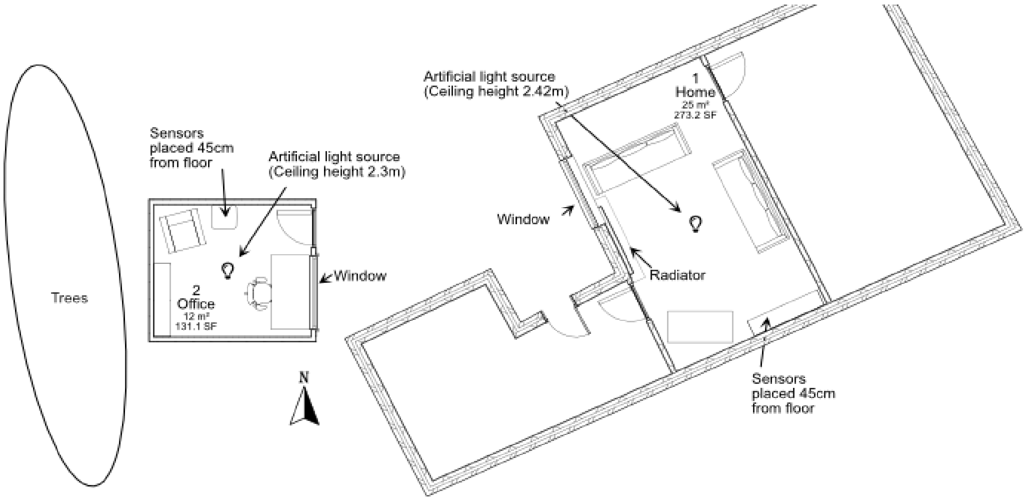

Supplementary Material Figure S2. Outline of the VoiceFlow application showing the visual script used to create the Alexa Skill

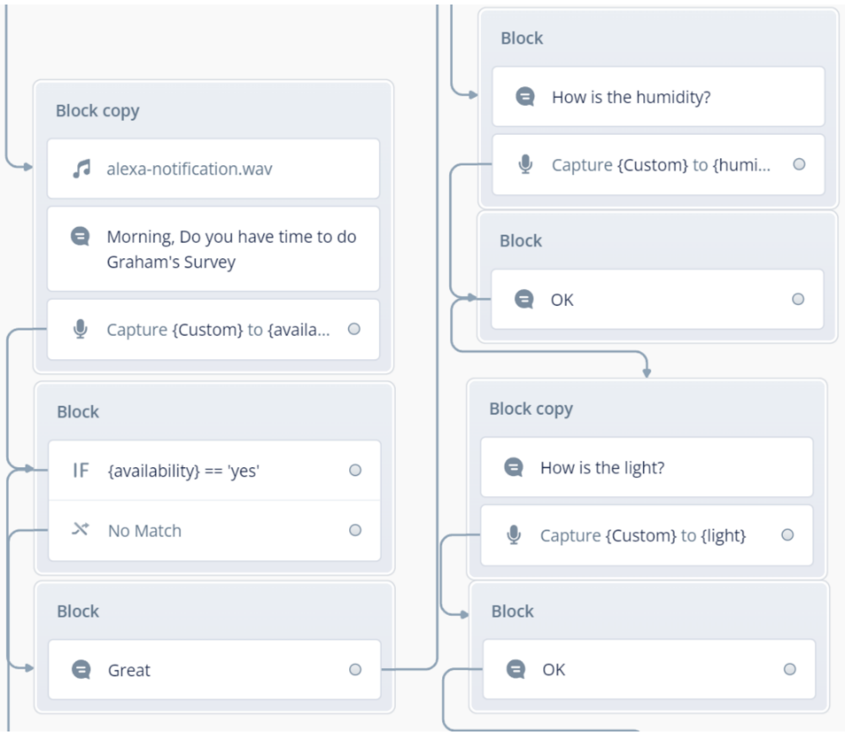

Supplementary Material Table S1. Descriptive statistics for quantitative data

	N	Minimum	Maximum	Mean	Std. Deviation
**HOME_TEMP** ***(°C)***	168849	16.85	32.36	25.10	1.81
**HOME_RH** ***(%)***	168785	25.30	59.00	41.44	4.89
**HOME_LIGHT** ***(lx)***	168851	0.00	650.00	10.72	31.29
**HOME_SOUND** ***(dBA)***	168846	34.30	87.42	39.09	3.05
**HOME_CO2** ***(ppm)***	168851	390.00	1,317.00	572.27	141.20
**HOME_eCO2** ***(ppm)***	168851	400.00	7,992.00	1,187.73	1,111.21
**HOME_PM25** ***(μg/m3)***	168851	0.00	212.00	1.10	4.84
**OFFICE_TEMP** ***(°C)***	154396	3.64	42.17	22.02	6.57
**OFFICE_RH** ***(%)***	154362	13.43	57.95	36.00	5.38
**OFFICE_LIGHT** ***(lx)***	154396	0.00	26,460.00	177.40	839.41
**OFFICE_SOUND** ***(dBA)***	122532	24.86	109.62	45.23	15.17
**OFFICE_CO2** ***(ppm)***	154396	258.00	2,644.50	618.18	191.85
**OFFICE_eCO2** ***(ppm)***	154396	400.00	7,992.00	595.56	459.93
**OFFICE_PM25** ***(μg/m3)***	154396	0.00	141.00	0.89	2.38
**Outdoor Humidity (%)**	205016	21.00	100.00	77.00	15.45
**Outdoor Temp (°C)**	205016	-2.89	27.01	10.70	5.42
**Air Quality Index**	205016	1.00	4.00	1.42	0.57
**Step Count**	205016	0.00	218.00	5.78	18.75
**Heart Rate**	205016	39.00	160.00	65.435	11.21

Supplementary Material Figure S3. Randomly selected sun map for the study location to give an idea of where the sun (yellow circle) would be throughout the day in relation to the buildings and rooms.

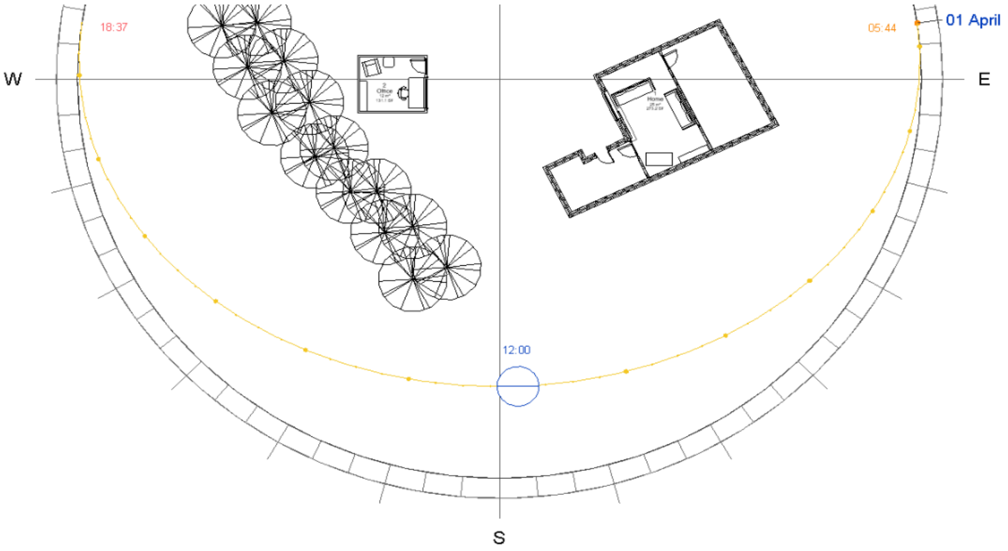

Supplement: Supplementary file 1 [file ijerph-20-04897-s001.zip › ijerph-2219297-supplementary.pdf]
